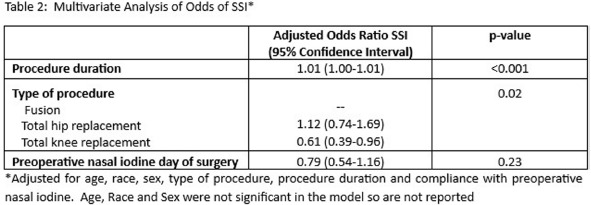# Evaluation of Risk Factors for Fungal Infections Post Cardiac Surgery: a Single Center Study

**DOI:** 10.1017/ash.2025.408

**Published:** 2025-09-24

**Authors:** Johanna Brait, Majd Alsoubani, Gabriela Andujar-Vazquez

**Affiliations:** 1 Massachusetts General Hospital; 2 Tufts Medical Center; 3 Dartmouth Hitchcock Medical Center

## Abstract

**Background:** Invasive candida infections (ICI)are rare but a serious complication following cardiac surgery, The incidence of ICI ranges between 1-2%. There are a few studies describing the risk factors associated with candidal infections in this population. This study aims to evaluate the risk factors of ICI post-cardiac surgery. We hypothesize that judicious antimicrobial use and comprehensive wound care play a key role in prevention of ICI. **Methods:** We conducted a retrospective case control study of adult patients undergoing cardiac surgeries at an academic medical center from January 2023 to June 2024. Patients who underwent heart transplantation were excluded. For each case, four controls who underwent similar surgical procedures, two before and two after the cases, were selected. ICI was defined as the detection of candida species by culture or histological examination from a normally sterile site like candidemia or mediastinitis. Cardiac surgery included valve replacement, coronary artery bypass graft and durable cardiac device insertion. Data were analyzed for demographics, type of surgery, temporary mechanical circulatory support (MCS) use and timing, chest tube duration, tracheostomy, dialysis and Candida sp, colonization, defined as the isolation of candida sp. in the urine or airways without evidence of infection. Categorical and continuous variables were presented as frequencies and medians respectively. The variables were compared using Chi-square and Mann-U-Whitney. **Results:** There were 36 controls, and 9 cases included in the study. Patients who were younger (54 vs 66.5 years) and who had temporary MCS (66.7% vs 8.0%) were more likely to be diagnosed with ICI. Moreover, we found that delayed chest closure, more days with chest tube in place, dialysis, tracheostomy and candida colonization after surgery were also associated with increased risk of ICI (table). However, antimicrobial use prior to surgery was not statistically significant (72.2% vs. 88.9%) In terms of clinical outcomes, there was no statistical difference in mortality between the two groups (66.7% vs 91.7%), however patients were more likely to have longer length of hospital stay (42 vs 11 days, p=0.03). **Conclusion:** This study identified several risk factors for ICI post-cardiac surgery including temporary MCS use, delayed chest closure, prolonged chest tube placement and tracheostomy. While antibiotic use prior to surgery was not statistically significant, candida colonization post-surgery was identified as a risk factor. These findings highlight the importance of infection prevention strategies in the environment of care, such standardizing temporary MCS device care and optimizing wound care management, as

**Figure:**
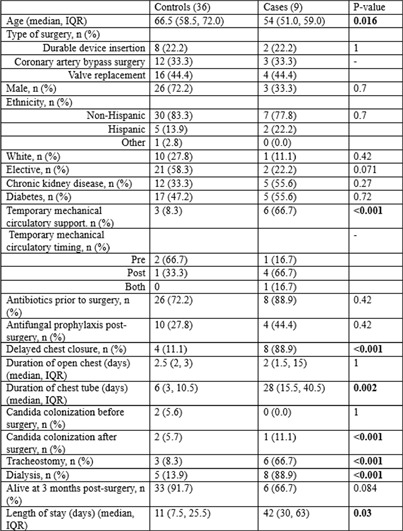

**Figure:**
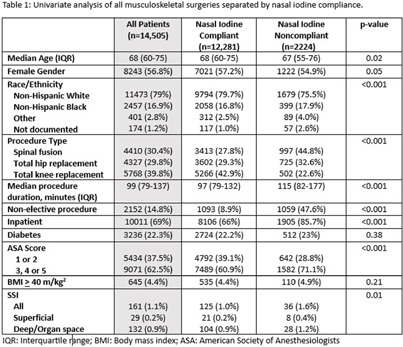

**Figure:**